# Digital health: The answer to the perfect storm negatively impacting the evolution of wound care specialisation

**DOI:** 10.1111/iwj.13991

**Published:** 2022-10-28

**Authors:** Douglas Queen

As wound carers most of us understand the impact of practice variation in the provision of wound care. Today we generally see sub‐optimal outcomes in most geographies around the world. This is not surprising since care is provided across many differing clinical settings and by many different clinical providers.^1^ This lack of specialisation provides a lack of consistency with variation in practice and outcomes, which was greatly exacerbated during the COVID pandemic.^2^

Several factors can influence this inconsistency in practice:

- with currently around only 40% of wound care specialists regularly using assessment tools, poor patient outcomes routinely result due to this inconsistency^3^;
- with over 25% of wounds having no recorded differential diagnosis^4^;
- with prolonged referral times, often resulting in a breakdown in continuity of care^5^;
- with a lack of risk profiling due to infrequent and inconsistent care^6^;
- with a lack of training and the emerging global health practitioner shortage^7^, ^8^;

we have the perfect storm to stall the evolution of wound care as a specialty.^9^

Digital health, thanks to COVID, seems to be a recent buzz in health care. However, it is not recent and has existed for a couple of decades—think EHR's.^10^ By 2021 there were over 350 000 health care apps, with 90 000 added last year alone.^11^ That is a whopping 250 per day! So, the development of digital health is accelerating for sure and perhaps the buzz is real after all. But as always in wound care, the growth of digital health has been slow, to say the least. However, as with other clinical areas, the recent pandemic has changed the evolutionary trajectory for all health care providers, making digital health a growing part of care.

A recent study^11^ demonstrated that currently:

- 56% of practitioners rely on apps to make decision in their practice;
- 39% believing they will increase efficiency, saving time;
- 36% believe digital health improves their interactions with their patients;
- 61% of patients living with a chronic condition use a health care app;
- 14% however only use the app for their specific chronic condition.

So, what are apps? Apps are essentially little bits of software that allow a specific task, to meet a specific purpose. Currently health care practitioners (HCPs) believe that the benefits of apps in general occur in the areas of patient engagement, access to care and patient safety, while providing convenience, efficiency and accuracy in the management of patients. Clinicians have stated that they currently use apps for admin, to manage their time, communicate with others, share and retrieve information, to receive and deliver education and training, to aid diagnosis, monitor their patients and of course within electronic health records where apps and digital health can help significantly.

In a 2020 survey by HealthDay/Harris Pool Survey:

- 46% of HCPs stated that they would be introducing an app into their practice;
- 50% of HCPs doing so to increase efficiency;
- 72% of HCPs surveyed believed that an app would encourage patients to be more engaged and responsible for their own health;
- 86% of HCPs believe apps will increase their patient's knowledge.

So, while we may have the perfect storm potentially negatively impacting the evolution of wound care as a speciality, we may have the perfect solution in digital health.^10^
